# Probing Changes in Ca^2+^-Induced Interaction Forces between Calmodulin and Melittin by Atomic Force Microscopy

**DOI:** 10.3390/mi11100906

**Published:** 2020-09-30

**Authors:** Sheng Huang, Jianhua Wang, Heng Sun, Yuna Fu, Yan Wang

**Affiliations:** Key Laboratory of Biorheological Science and Technology, Ministry of Education College of Bioengineering, Chongqing University, Chongqing 400044, China; stevenhouse@cqu.edu.cn (S.H.); sunheng@cqu.edu.cn (H.S.); fuyuna@cqu.edu.cn (Y.F.); wangyan1992@cqu.edu.cn (Y.W.)

**Keywords:** mechanobiology, calmodulin, melittin, atomic force microscopy, self-assembled monolayer

## Abstract

Mechanobiology studies the means by which physical forces and mechanical properties change intra- or inter- biological macromolecules. Calmodulin (CaM) is involved in physiological activities and various metabolic processes in eukaryotic cells. Although the configuration changes in the interaction between calmodulin and melittin have been studied, the biomechanical relationship of their interaction has rarely been explored. Here, we measured the adhesion forces between calmodulin and melittin in solutions of gradient concentration of calcium ions using atomic force microscopy (AFM). We found that the specific (*F_i_*) and nonspecific (*F_0_*) adhesion forces between single melittin and calmodulin in a PBS solution were 69.4 ± 5.0 and 29.3 ± 8.9 pN, respectively. In the presence of 10^−7^ to 10^−3^ M Ca^2+^ PBS solution, the *F_i_* increased significantly to 93.8 ± 5.0, 139.9 ± 9.0, 140.4 ± 9.7, 171.5 ± 9.0, and 213.3 ± 17.8 pN, indicating that the unbinding force between melittin and calmodulin increased in the presence of Ca^2+^ in a concentration-dependent manner. These findings demonstrated that biomechanical studies based on AFM could help us better understand the melittin/calmodulin-binding processes in the presence of calcium and help us design and screen peptide drugs based on calmodulin.

## 1. Introduction

Mechanobiology is an emerging research field with the primary purpose of linking biological and mechanical engineering, which has attracted increasing attention and participation from researchers in recent years [1,2]. One branch of this domain is focused on studying the means by which physical forces and mechanical properties change intra- or inter- biological macromolecules [3,4,5]. The reason is that protein-based receptor–ligand interactions [6,7,8] and protein folding [9,10] activities are known to run through the entire biological processes, and changes in the mechanical properties of these processes play a crucial role [11,12,13]. Many technologies have emerged to explore the interaction between or within biological macromolecules, such as biomembrane force probes (BFPs) [14,15], optical tweezers [16,17], microneedle manipulation [18,19], magnetic tweezers [20,21], and atomic force microscopy (AFM) [22,23,24]. Among these evolving technologies, AFM has shown increasing advantages due to: (1) the fact that it can be operated with high force sensitivity and obtained nanometric morphology resolution [25,26]; (2) its ability to simulate the measurement of intermolecular or intramolecular interactions under physiological conditions, such as receptor–ligand interactions and protein folding, which might help explain some essential pathogenic mechanisms [27,28]; (3) its ability to participate in the study of the adhesion [29], elasticity [30], dynamics process [31], and other characteristics of biological samples, and to quantitatively analyze the protein–protein interaction, as well as to elaborate the nature and magnitude of the interaction force between biomolecules and the related binding energy properties [6,32].

**Figure 1:**
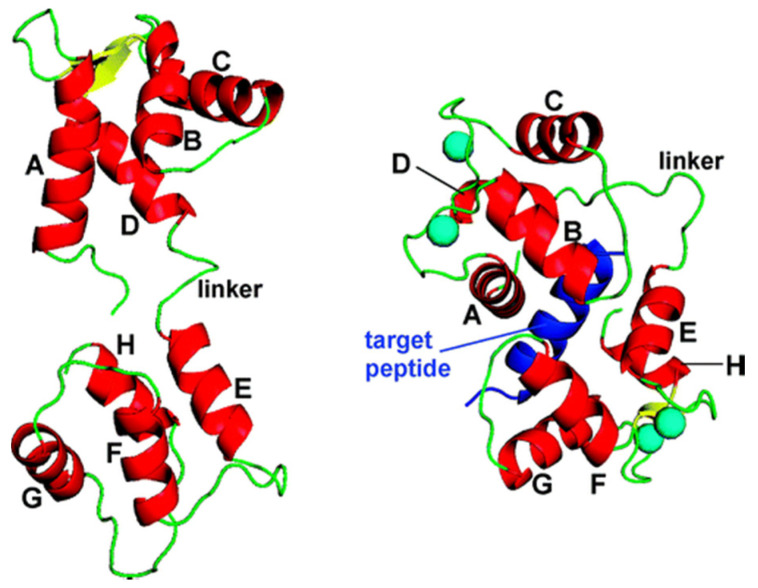
Three-dimensional (3D) structure of apo-CaM (PDB code 1CFD (3), (**left**)) and an example of a canonical Ca4 3 CaM 3 target complex (PDB code 1CM1 (18), (**right**)). CaM helices A–H are shown in red, and the target peptide is depicted in dark blue. Cyan-colored spheres represent calcium ions [37].

Calmodulin (CaM) is a highly conserved protein and is ubiquitously found in eukaryotic cells [33]. More specifically, CaM, which is present on the surface of eukaryotic cell membranes, is known to play a unique regulatory role in physiological activities and various metabolic processes, such as protein phosphorylation/dephosphorylation [34], cell division [35], and cell apoptosis [36]. As a primary receptor of Ca^2+^ ion, it can be reversibly adopt an inactive (apo-CaM, Ca^2+^-free calmodulin) or active (holo-CaM, Ca^2+^-saturated calmodulin) configuration following changes in the intracellular concentration of calcium, thereby binding to multiple enzyme targets within the cell to mediate various biological activities in the organism. (The three-dimensional structure of CaM is shown in Figure 1).

**Figure 2:**
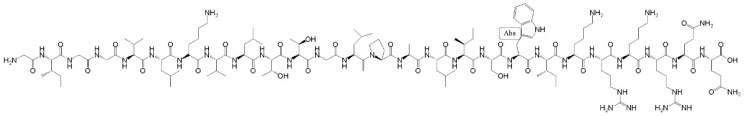
The structure of melittin.

Melittin (MEL) is known to be a significant peptide in bee venom; its structure is shown in Figure 2. Melittin consists of 26 amino acids and exhibits many excellent biological activities, such as antibacterial, antiviral, and anticancer functions [38]. Through in-depth mechanistic research, it was found that melittin could interact with various proteins, such as protein kinases [39] and phospholipases [40]. Melittin has been reported to participate in the regulation of multiple biological reactions by altering the activity of proteins and enzymes through interaction with these molecules and has also been shown to exhibit or activate various biological activities.

Calcium is a critical element of the body of life and is involved in various biological functions. The cell transports extracellular calcium ions into the cell through calcium-binding proteins and calcium pumps on the membrane surface. The calcium ion signal generated in this process acts as a second messenger to regulate multiple signal pathways for life activities [41]. Calmodulin (CaM) is the essential calcium-binding protein in the body and is involved in regulating many calcium-dependent signaling pathways [42]. Melittin is one of the most effective calmodulin inhibitors. When it binds to calmodulin, it can cause cancer cell death. The inhibition of calmodulin activity has been shown to inhibit DNA synthesis and tumor cell growth. The research results from Lazo et al. [43] showed that bee venom peptide could cause DNA damage and cytotoxicity to leukemic L1210 cells based on the competitive inhibition of calmodulin. Therefore, an in-depth study of the interaction between melittin and calmodulin helps elaborate on the mechanism of anticancer activity of melittin based on calmodulin and the development of anticancer drugs based on melittin in the future.

A variety of approaches are available to probe the structure, composition, and properties of the calmodulin–melittin system, including isothermal titration calorimetry (ITC) [44], Fourier transform ion cyclotron resonance mass spectrometry (FTICR-MS) [45], surface-enhanced infrared absorption spectroscopy (SEIRAS) [46], and Fourier transform infrared spectroscopy (FTIR) [47]. Changes in the configuration and binding sites might only explain the interaction between melittin and calmodulin at a specific time and space. However, in a natural physiological environment, the interaction between melittin and calmodulin is a continuous dynamic process. Therefore, to further study the structure–activity relationship between melittin and calmodulin, research on their interaction forces, whether in the absence or presence of calcium ions, should be conducted because the protein–ligand interaction force plays a critical role in the biological system processes. Besides, in our limited cognitive scope, research has never focused on studying the interaction force between calmodulin and melittin in different environments.

In this study, we employed a self-assembled monolayer (SAM) method to fix calmodulin on a gold substrate and melittin on the surface of the gold modified tip. The SAM method for preparing AFM samples has been demonstrated to be simple and effective in our previous research. Accordingly, we used AFM to measure the changes in specific and nonspecific interactions between calmodulin and melittin in the presence or absence of Ca^2+^ ions. The results showed that calcium ions significantly affected the mechanical interaction between calmodulin and melittin. Also, the specific interaction force between melittin and calmodulin increased with the increase of the calcium concentration.

## 2. Materials and Methods

### 2.1. Materials

N-hydroxysuccinimide (NHS), N-(3-dimethylaminopropyl)-N-ethylcarbodiimide hydrochloride (EDC), sulphuric acid(H_2_SO_4_) (ACS reagent), Hydrogen peroxide solution (H_2_O_2_) (34.5–36.5%), and 16-mercaptohexadecanoic acid (MHA) were purchased from Sigma-Aldrich (Sigma-Aldrich Shanghai Trading Co Ltd., Shanghai, China) and used as received. Furthermore, 1 × PBS (pH 7.2–7.4, 0.01 M, cell culture) was obtained from Thermo Fisher Scientific (Shanghai, China), calcium chloride and ethanol (MS grade) were purchased from Merck (Merck KGaA, Darmstadt, Germany). Calmodulin isolated form bovine heart (lyophilized powder, 2500–10,000 units/mg protein) and melittin were purchased from Sigma-Aldrich (Sigma–Aldrich Shanghai Trading Co Ltd., Shanghai, China). Deionized water (18.25 MΩ·cm) was obtained in-house using a Direct-Q 3 Millipore Ultra-pure water purification system (Millipore, Burlington, MA, USA).

### 2.2. Preparation of Gold Substrate

In this experiment, a tweezer was used to gently peel off a single layer of mica in a sterile operating box. The mica was immediately removed into the radiant tube heater and heated for 2 h at 325 °C. Then, the preheated mica was transferred to a super-vacuum evaporator to prepare the gold-coated substrate by the vapor deposition method at approximately 10^−7^ Torr. The evaporation rate was controlled to 0.1–0.3 nm/s, and the final thickness of the gold layer was identified to be approximately 200 nm. In addition, a chromium film was deposited in mica before the gold to increase the adhesion force of the gold molecule on mica. Finally, the gold-coated mica was annealed for 1 min by H_2_ before use.

### 2.3. Self-Assembled Monolayer (SAM) of Thiol on Gold Surfaces

The prepared bare gold-coated substrate was carefully cleaned with a hot piranha solution (*v/v*, H_2_O_2_: H_2_SO_4_ = 1:3) for 30 min to remove any organic matter present on the surface. After that, the gold substrate was alternately washed with ethanol and ultra-pure water 3 to 5 times. The surface of the gold substrate was then dried with high purity nitrogen gas and dipped in an ethanol solution of 1 mM MHA for 24 h. The terminal of the mercaptan of MHA is known to bind to the surface of the gold substrate by covalent binding to form a self-assembled monomolecular layer. Subsequently, the gold substrate was placed in ethanol for ultrasonic cleaning for 2 min to remove unlinked thiols. Finally, the thiol-based SAM was alternately rinsed with ultra-pure water and ethanol 3 to 5 times and then dried with nitrogen.

### 2.4. Protein Immobilization onto the Gold Surfaces

Calmodulin was fixed to the gold substrate by forming amide bonds through its free amino groups and the carbonyl groups of MHA. First, the carbonyl groups needed to be activated. In this operation, the thiol-based SAM was immersed in 2 mg/mL NHS and 2 mg/mL EDC of a PBS solution for 1 h at 25 °C. The thiol-based SAM was washed 3 to 5 times with ultra-pure water and dried with high-purity N_2_. Then, the activated SAM was placed in 10 mM of calmodulin-containing PBS solution for 12 h at 4 °C. The prepared SAM was then sequentially rinsed with pure ethanol and ultra-pure water 3 to 5 times. Finally, the prepared calmodulin-immobilized-containing SAM was stored in PBS solution at 4 °C before use. The matrix-immobilized calmodulin samples were used within 2 d.

### 2.5. Functionalization of Atomic Force Microscopy (AFM) Tip

A functionalized gold-modified tip with melittin was prepared as described above, except the tip was immersed in a melittin-containing PBS solution.

### 2.6. Measurement of the Melittin-Calmodulin Adhesion Force

The adhesion force between melittin and calmodulin was measured using Park systems NX-10 atomic force microscopy (Park system Co., Suwon, Korea). The melittin-functionalized AFM tip was first scanned across the calmodulin monolayer and bare gold substrate (as blank control) at a randomly selected location. Then, force measurements were taken with the melittin-functionalized tip moved toward the desired point of the surface of the calmodulin monolayer and retracted back to the initial set point. When the approached the monolayer surface and then retracted away from the binding point, it was deflected due to the melittin-calmodulin interaction force, which was detected on the instrument as a “voltage-displacement” signal, followed by its transformation into a “force-displacement” curve. Typically, the AFM tip is considered as an elastic probe. Therefore, the deflection of the tip was converted to the force (*F*) generated on it according to Hooke’s law, that is,

*F* = *k* × *d*

where *d* is the deflection of the tip in the measurement and *k* is the practical spring constant of the tip during the measurement. Generally, the *k* should be small in this AFM experiment to reduce the measurement noise. For this study, a gold-coated Si_3_N_4_ cantilever tip was used in all the force experiments, and the spring constant was calibrated using the thermal fluctuation method (0.07–0.4 N/m). The radius of the tip was approximately 25 nm, while the thickness of the gold layer was approximately 70 nm.

All interaction force measurements between melittin and calmodulin were performed using contact mode at 25 °C. Several hundred of force curves were collected: 2 sets of experiments (~300 force curves per set) were conducted on 2 different samples using 2 different tips. For the measurements of the adhesion force, PBS solutions without and with gradient concentrations of calcium ions were used. The gradient concentration of Ca^2+^ was set as 10^−7^, 10^−6^, 10^−5^, 10^−4^, and 10^−3^ M. A syringe was used to replace the PBS solution with different concentrations of calcium ions in the AFM liquid cell. The velocity of the tip during retracement was set to 0.3 μm/s. The adhesion forces were calculated and analyzed from the collected “force-displacement” curves using the XEI processing software program (Park system Co., Suwon, Korea). Six locations in the protein monolayer were randomly selected for this study. Measurements were taken approximately 50 to 60 times at each point to obtain a more accurate statistical analysis. The calmodulin-free gold substrate was used as the control group. The block study was conducted by measuring the interaction forces in the presence of free melittin in a PBS solution.

### 2.7. AFM Imaging

All images were obtained using a Park Systems NX-10 atomic force microscopy (Park Systems Co., Suwon, Korea). The AFM imaging study was performed at a scan rate of 1 Hz, with the image resolution being set to 512 × 512. Briefly, AFM was performed in non-contact mode in PBS employing a scanning size of 1000 × 1000 nm. Images of the calmodulin monolayer and the roughness of surfaces under different conditions were collected and analyzed using the XEI processing software program (provided by the manufacturer).

## 3. Results and Discussion

### 3.1. Probing the Ca^2+^ Effect in the Interaction Forces between Melittin and Calmodulin

This study focused on the revelation of the spatial configuration of CaM, with its interactions with calcium ions and target peptides being conducted in a homogenous solution system [48,49]. However, in this solution system, it would have been difficult to dynamically study the mechanobiological functional changes of calmodulin with the target peptide caused by the dynamic changes of calcium ions, unless calmodulin was anchored to the surface of a matrix.

In our previous research, we provided a stable and reliable SAM method for protein immobilization on a thiol-modified gold matrix with a relatively simple preparation of SAM [50]. Accordingly, we employed an identical SAM method to explore the interactions between melittin and calmodulin by forming an orderly melittin and calmodulin monolayer on the surface of the gold-modified AFM tip and the matrix.

**Figure 3:**
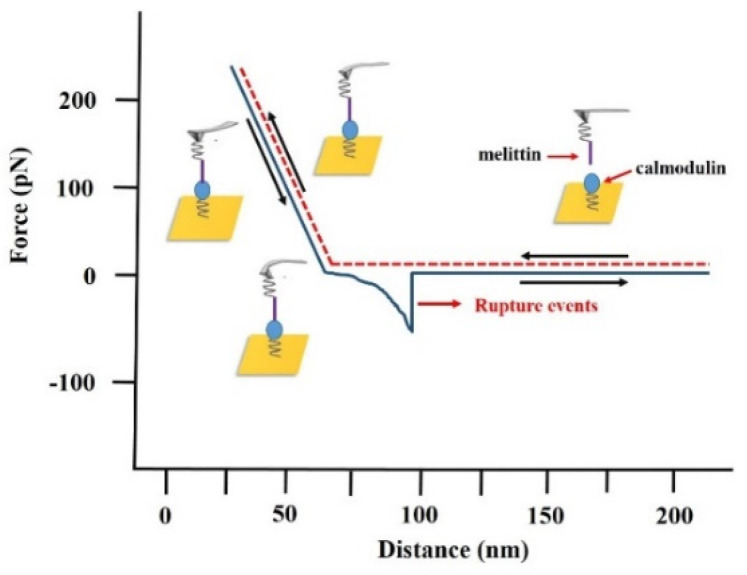
Example of the force–distance curve between melittin and calmodulin measured by atomic force microscopy (AFM). The extending stage is shown in the red dash line (probe approaches to a gold substrate); the retracting stage is shown in the solid blue line (probe tip withdraws from the gold substrate). Corresponding rupture events between melittin and calmodulin monolayer is also presented.

In this study, we measured the adhesion forces between melittin and calmodulin in different concentrations of Ca^2+^ solution calculated as rupture forces. In reality, the contact area between the melittin-modified tip and the calmodulin-fixed matrix was more than a single protein–peptide interaction. As a result, during a single extend and retract measurement, we collected a set of interaction forces generated between multiple pairs of melittin and calmodulin. The force–distance curve could thus show in facticity the interaction force between melittin and calmodulin. A typical approach and retracting process, and the probe force–distance curves between melittin and calmodulin are shown in Figure 3.

The adhesion event between melittin and calmodulin was observed to occur at the sharp point in the retraction curve, where the tip of the probe retreated to the critical point of contact with the matrix. The deflection of the AFM tip was hence converted to a specific value of the interaction force according to Hook’s Law:

*F* = *k* × *d*,

where *k* and *d* are the force constant and probe deflection, respectively.

**Figure 4:**
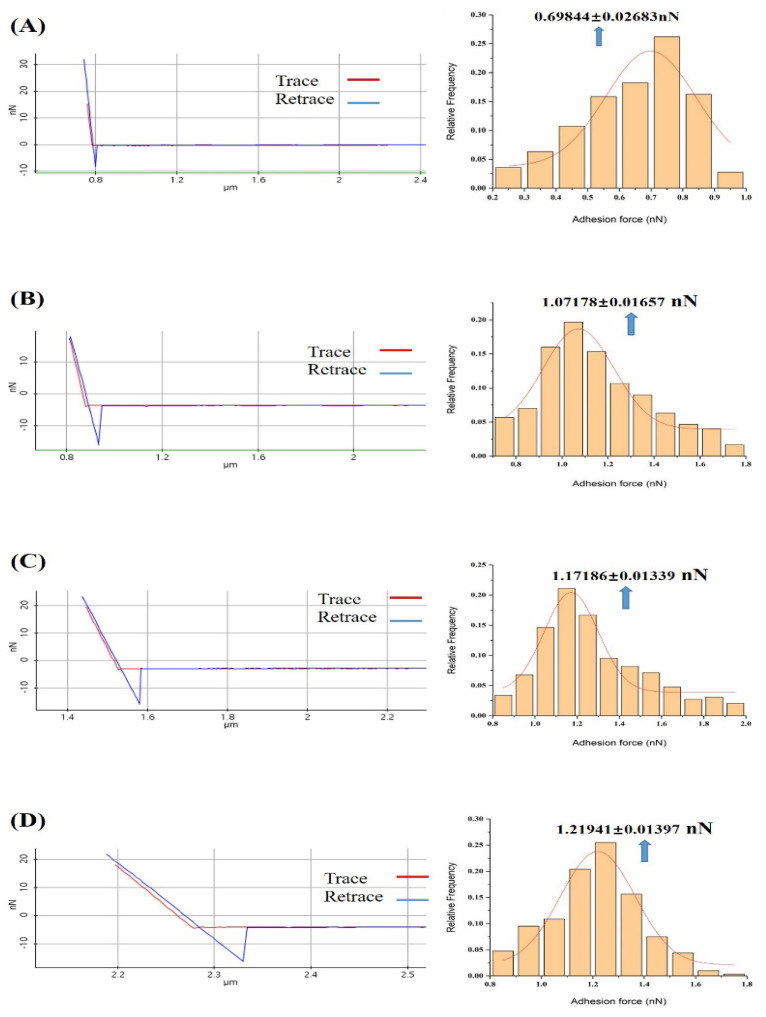

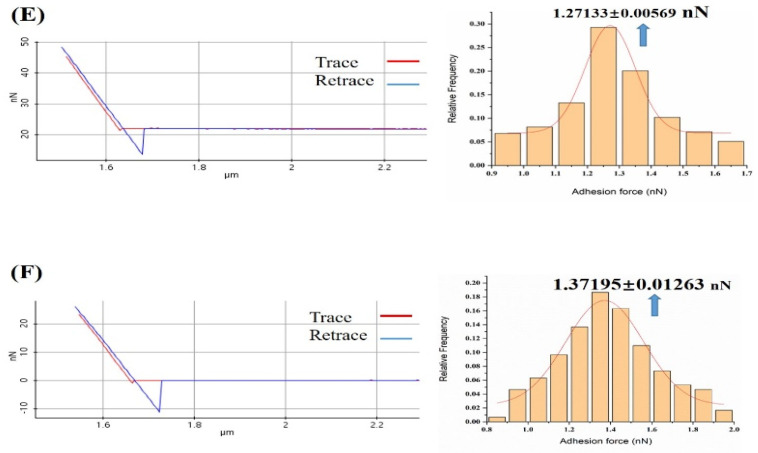
Representation of the force–distance curve and frequency distribution of all measured adhesion forces in the absence and presence of gradient concentration of calcium ions solution: (**A**) The probability distribution of measured adhesion forces in PBS solution; (**B**) The probability distribution of measured adhesion forces in 10^−7^ M concentration of calcium ions in the PBS solution; (**C**) The probability distribution of measured adhesion forces in 10^−6^ M concentration of calcium ions in the PBS solution; (**D**) The probability distribution of measured adhesion forces in 10^−5^ M concentration of calcium ions in the PBS solution; (**E**) Probability distribution of the measured adhesion forces in 10^−4^ M concentration of calcium ions in PBS solution; (**F**) Probability distribution histograms of measured adhesion forces in 10^−3^ M concentration of calcium ions in PBS solution. Nonlinear fitting to Gaussian models of the distributions is shown.

We measured the adhesion forces between melittin and calmodulin in the absence and presence of a gradient concentration of calcium ions in a PBS solution. We then calculated the probability distributions from repeated force measurements (Figure 4) and generated a histogram of measured adhesion forces in the PBS solution from near 300 force–distance cycles. We consecutively fitted the distribution of the adhesion forces between melittin and calmodulin obtained in the absence and presence of a gradient concentration of calcium ions solution to Gaussian models. The Gaussian distribution revealed the distribution value of the adhesive force measured by random selection points. Similar results have been obtained in previous studies in our laboratory; that is, Gaussian fitting was used to elucidate the adhesion force values between protein molecules at the single molecular level [51,52].

The frequency distribution histogram and Gaussian fitting curve of the adhesion between melittin and calmodulin showed a peak in the PBS solution (Figure 4A), with the peak values being presented as 0.70 ± 0.026 nN. We observed the presence of markedly elevated peaks (Figure 4B–F) in the presence of 10^−7^ to 10^−3^ M Ca^2+^ PBS solution, with a peak value of 1.07 ± 0.017, 1.17 ± 0.013, 1.22 ± 0.014, 1.27 ± 0.006, and 1.37 ± 0.012 nN, respectively.

### 3.2. Effect of Ca^2+^ Concentration on Specific and Nonspecific Forces

We also noted that during the actual process, the adhesion measured by AFM was not the interaction between single molecular melittin and calmodulin, but the result of the interaction between multiple melittin and calmodulin pairs. As such, we used the SAM method to modify the AFM tip. The tip had a certain radius of curvature, that is, there were more than a single melittin molecule on the tip and multiple calmodulin molecules on the substrate were therefore reflected in the adhesion value obtained by the AFM measurement. To resolve this, we applied the Poisson method [53,54] to analyze the unbinding force needed to separate a single pair of melittin and calmodulin.

In Poisson distribution, the probability *P(X)* of random variable *X* equal to non-negative integer value *μ* is as follows:

$$P\left(X\right)=\frac{e^{-\mu }\mu^{x}}{X!}$$

The sum of probabilities is 1, that is:

$$\overset{\infty }{\underset{X=0}{\sum }}P\left(X\right)=1$$

Therefore, the probability of *X* = 0 event is as follows:

$$P\left(X=0\right)=\frac{e^{-\mu }\mu^{0}}{0!}=e^{-\mu }$$

Except 0, the total probability of other events is:

$$P\left(X\geq 1\right)=1-P\left(X=0\right)=1-e^{-\mu }$$

The mean value and variance of the system can be calculated by the following formula:

$$\mu =\overset{\infty }{\underset{X=0}{\sum }}XP\left(X\right)=\overset{\infty }{\underset{X=0}{\sum }}XP\left(X\right)+0P\left(0\right)=\overset{\infty }{\underset{X=0}{\sum }}XP\left(X\right)$$

$$\sigma^{2}=\overset{\infty }{\underset{X=0}{\sum }}{\left(x-\mu \right)}^{2}P\left(X\right)=\overset{\infty }{\underset{X=1}{\sum }}(X^{2}-2\mu X+\mu^{2})P\left(X\right)+\mu^{2}e^{-\mu }$$

According to the definition of Poisson distribution, the mean value and variance of *n* pairs of action bonds are equal:

$$\mu_{n}=\delta_{n}^{2}$$

The adhesion force (*F*) measured from the force distance curve is related to the bond fracture number (*n*) in a single pull-off event:

$$F=nF_{i}$$

Here, *F* represents the mean value of the single bond breaking force in the system, which is expected to be a constant value. The mean ($\mu_{m}$) and variance ($\sigma_{m}^{2}$) value of the pull-out force can be obtained by measuring multiple pull-out events. Based on the relationship between the measured force and the fracture number, the following formula is given:

$$\mu_{m}=\mu_{n}F_{i}$$

$$\sigma_{m}^{2}=\sigma_{n}^{2}F_{i}^{2}$$

Then the fracture force ($F_{i}$) between single pair of bonds can be calculated by the measured value:

$$F_{i}=\frac{\sigma_{m}^{2}}{\mu_{m}}$$

When the possible nonspecific action (*F_0_*) is considered, the calculation formula of the system is changed as follows:

$$\mu_{m}=\mu_{n}F_{i}+F_{0}$$

$$\sigma_{m}^{2}=\sigma_{n}^{2}F_{i}^{2}=\mu_{m}F_{i}-F_{i}F_{0}$$

Based on the Poisson model, we can derive the specific unbinding force between the possible nonspecific interaction force (*F_0_*) and a single melittin-calmodulin pair (*F_i_*) can be derived from the following equation:

$$\sigma_{m}^{2}=\mu_{m}F_{i}-F_{i}F_{0}$$

where $\sigma_{m}^{2}$ and $\mu_{m}$ represent the variance and the mean of the measured total adhesion force, respectively.

**Figure 5:**
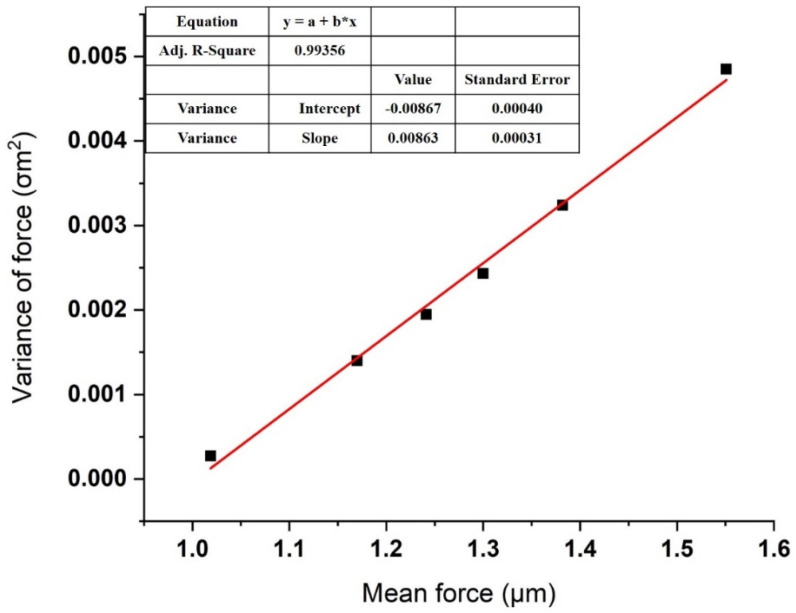
Linear fitting of the variance plotted versus the mean of measured adhesion force in the presence of Ca cation. Each point represents a dataset taken at one of the six randomly selected locations. Details of the dataset are given in Table 1.

We obtained the total interaction forces between melittin and calmodulin by repeatedly measuring them for 50 to 60 times at each of 6 several randomly chosen points of the calmodulin monolayer in the PBS solution with gradient concentrations of calcium ions (10^−7^, 10^−6^, 10^−5^, 10^−4^, and 10^−3^ M). The mean ($\mu_{m}$) and variance ($\sigma_{m}^{2}$) of the total 300 times pull-off event measured in the PBS solution are shown in Table 1, while the linear fitting of the plotted versus the variance is presented in Figure 5.

**Figure 6:**
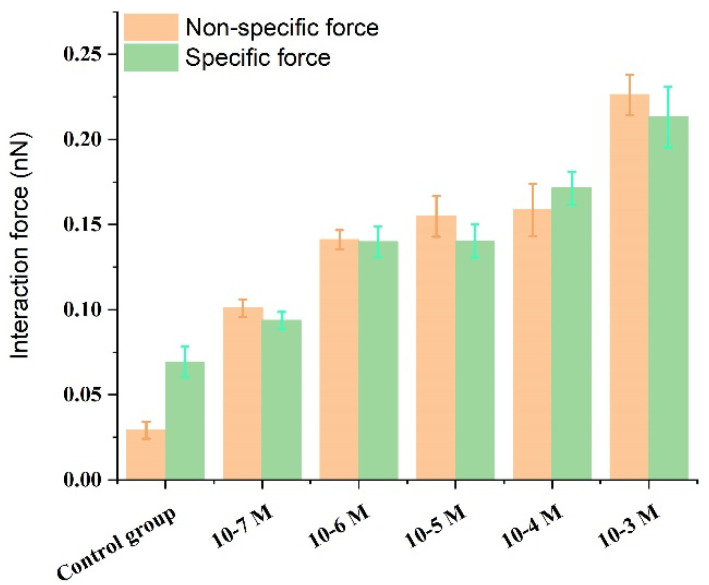
Bar representation presenting specific/nonspecific forces between melittin and calmodulin in the control group and Ca^2+^ concentration-dependent groups.

**Figure 7:**
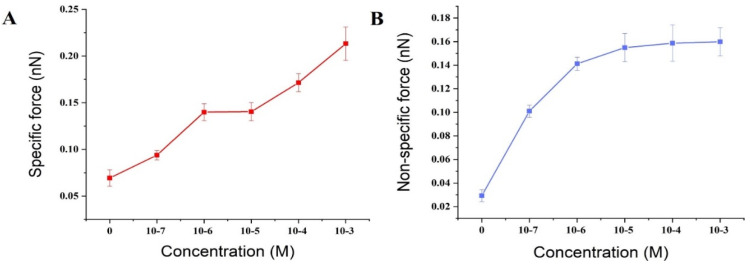
The specific and nonspecific forces between melittin and calmodulin with changing concentrations of Ca^2+^: (**A**) the specific forces changing between melittin and calmodulin with increased concentrations of Ca^2+^; (**B**) the non-specific forces changing between melittin and calmodulin with increased concentrations of Ca^2+^.

The activation by calcium ions and binding to target proteins/polypeptides are known to be the main mechanisms of the CaM-mediated regulation of proteins. Most of the target peptides that bind with CaM are known to be amphipathic, basic, and with α-helical structures. Melittin, as a typical pattern peptide, has been widely used in studies of CaM activity. Earlier studies have found that the peptide binds strongly to calcium-saturated calmodulin at a molecular ratio of 1:1 [55], but much less strongly to apo-CaM [56]. It has been hypothesized that calmodulin saturated with calcium ions binds to the C-terminal of melittin through its C-terminal binding domain [57]. The above conclusions could support our experimental results. The experimental results of this study showed that in a PBS solution without calcium ion, there was a small specific interaction between fixed calmodulin and melittin with a value of 69.4 ± 8.9 pN. It is suggested that melittin can also interact with calmodulin in the absence of calcium ion, which verifies the research results of Itakura et al. [58]. Whereas in the presence of calcium ions and an increase in their concentration, the specific interaction between calmodulin and melittin was observed to gradually increase to 93.8 ± 5.0, 139.9 ± 9.0, 140.4 ± 9.7, 171.5 ± 9, and 213.3 ± 17.8 pN (Figure 6). Moreover, an early study found that apo-CaM binding to Ca^2+^ led to structural transformation resulting in gradual exposure of the hydrophobic domain of CaM [59]. These increase hydrophobic parts of CaM enabled the Ca^2+^-binding of CaM is easier to bind closely with melittin which leads to a gradual increase in the specific interaction between melittin and calmodulin (Figure 7A).

In addition, as the chemical and hydrogen bonds are considered specific interactions, whereas electrostatic interactions are included in nonspecific interactions in the Poisson distribution analysis, an increase in the concentration of calcium leading to a change of ion strength in solution would be expected to result in an increase in the nonspecific interactions between melittin and calmodulin (Figure 7B). This result implies that calcium ions could participate in the melittin and calmodulin-binding behaviors and accelerate the conformational transition of calmodulin [60].

### 3.3. Calmodulin Imaged by AFM in Solution

**Figure 8:**
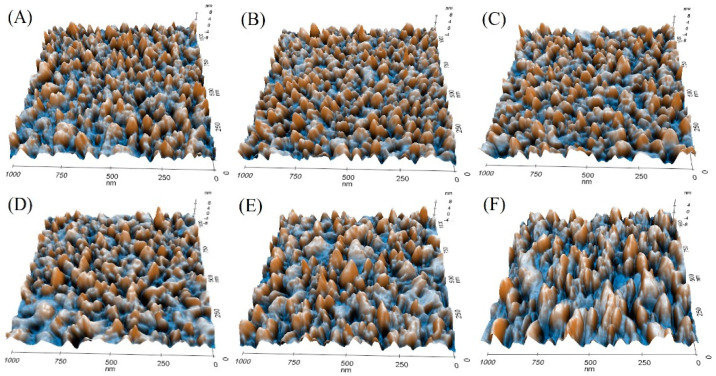
3D topography of calmodulin imaged by AFM in PBS solution (**A**) and in 10^−7^, 10^−6^, 10^−5^, 10^−4^, and 10^−3^ M Ca^2+^ PBS solution (**B**–**F**).

In order to evaluate the potential effect of the concentration of calcium ions in the structure of calmodulin, we detected and subsequently analyzed the monolayer morphology of calmodulin in a PBS solution with different concentrations of calcium ions. As shown in Figure 8, the calmodulin monolayer was clearly arranged in the PBS solution with apparent boundaries. The image showed the shape of a small, rounded particle. In contrast, calmodulin imaging revealed an increased grain size, irregular boundary, and reduced clarity in the calcium ion solutions of gradient concentration.

Using software analysis, we found that the roughness of the calmodulin monolayer was changed. The roughness of the topography was integrated and characterized by an average roughness (Ra) (Table 2). The results showed that the average roughness of calmodulin in PBS solution without calcium ion was 1.616 nm. With the addition of calcium ions and the increase of concentration, calmodulin gradually combined with calcium ion and changed its conformation. The aggregation of calmodulin particles was observed from the 3D conformation diagram (Figure 8), which resulted in the increase of average roughness of calmodulin monolayer on the gold substrate (1.641, 1.789, 1.844, 1.849, 1.858 nm). This is because when the ion concentration of the solution increases, the water interaction of the protein is weakened, and the electrostatic bimolecular layer on the surface of the protein is compressed, which makes the electrostatic interaction between protein molecules strengthen, leading to the aggregation of protein molecules [61]. It is also proved that the concentration of calcium ion can affect the conformational change of calmodulin [37].

## 4. Conclusions

In summary, calmodulin was fixed to a gold surface matrix using a convenient and straightforward self-assembly method. The interaction between calmodulin and its inhibitor melittin in a PBS solution with and without gradient concentration of calcium ions was explored at the single molecular level. Our results showed that the specific interaction between calmodulin and melittin was gradually increased with the presence and increase in the concentration of calcium ions. In addition, we also evaluated the morphology of calmodulin in the absence or presence of varying concentrations of calcium solutions. Our results indicated that the unbinding force between melittin and calmodulin was increased in the presence of a gradient Ca^2+^ PBS solution in a concentration-dependent manner. These findings demonstrated that biomechanical studies based on AFM could help us better understand the melittin/calmodulin-binding processes in the presence of varying concentrations of calcium ions. At the same time, our study further supported that AFM could be used as a powerful tool to assist in the design and screening of drugs related to the signaling pathways in which calmodulin is involved.

## Figures and Tables

**Table 1:** Unbinding forces between melittin and calmodulin measured in 10^–7^ M concentration of calcium ions in PBS solution at six randomly selected locations, repeated 50 to 60 times (*n* = 300).

Set	Mean, μm (nN)	$\mathrm{Variance},\;\sigma_{m}^{2}\;({\mathrm{nN}}^{2})$	Number of Bonds, μ_n_
1	1.01878	0.00027	7.4
2	1.1698	0.0014	8.7
3	1.24122	0.00195	11.6
4	1.3	0.00243	12.4
5	1.38184	0.00324	13.2
6	1.55082	0.00485	16.4

**Table 2:** Average roughness (Ra) of calmodulin layers in PBS solution with different concentrations of calcium ions.

Sample	Ra (nm)
The control	1.616
10^−7^ M Ca^2+^	1.641
10^−6^ M Ca^2+^	1.789
10^−5^ M Ca^2+^	1.844
10^−4^ M Ca^2+^	1.849
10^−3^ M Ca^2+^	1.858
